# Loop-mediated isothermal amplification assay for screening congenital cytomegalovirus infection in newborns

**DOI:** 10.1007/s00253-023-12771-2

**Published:** 2023-09-19

**Authors:** Hyeonseek Park, Doo Ri Kim, Areum Shin, Eunjung Jeong, Sohee Son, Jin-Hyun Ahn, So Yoon Ahn, Suk-Joo Choi, Soo-young Oh, Yun Sil Chang, Yae-Jean Kim, Minhee Kang

**Affiliations:** 1 Biomedical Engineering Research Center, Smart Healthcare Research Institute, Samsung Medical Center, Seoul, Republic of Korea; 2 Department of Medical Device Management and Research, Samsung Advanced Institute for Health Science & Technology, Sungkyunkwan University, Seoul, Republic of Korea; 3 Department of Pediatrics, Samsung Medical Center, Sungkyunkwan University School of Medicine, Seoul, Republic of Korea; 4 Department of Microbiology, Sungkyunkwan University School of Medicine, Suwon, Republic of Korea; 5 Department of Gynecology and Obstetrics, Samsung Medical Center, Sungkyunkwan University School of Medicine, Seoul, Republic of Korea; 6 Samsung Advanced Institute for Health Science & Technology, Sungkyunkwan University, Seoul, Republic of Korea

**Keywords:** Congenital cytomegalovirus infection, Polymerase chain reaction, Loop-mediated isothermal amplification, Newborn screening

## Abstract

**Abstract:**

Congenital cytomegalovirus (CMV) infection is a common cause of sensorineural hearing loss and neurodevelopmental impairment in newborns. However, congenital CMV infection cannot be diagnosed using samples collected more than 3 weeks after birth because testing after this time cannot distinguish between congenital infection and postnatal infection. Herein, we developed a robust loop-mediated isothermal amplification (LAMP) assay for the large-scale screening of newborns for congenital CMV infection. In contrast to conventional quantitative polymerase chain reaction (qPCR), which detects CMV within a dynamic range of 1.0 × 10^6^ to 1.0 × 10^2^ copies/μL, our quantitative LAMP assay (qLAMP) detects CMV within a dynamic range of 1.1 × 10^8^ to 1.1 × 10^3^ copies/μL. Moreover, the turnaround time for obtaining results following DNA extraction is 90 min in qPCR but only 15 min in qLamp. The colorimetric LAMP assay can also detect CMV down to 1.1 × 10^3^ copies/μL within 30 min, irrespective of the type of heat source. Our LAMP assay can be utilized in central laboratories as an alternative to conventional qPCR for quantitative CMV detection, or for point-of-care testing in low-resource environments, such as developing countries, via colorimetric naked-eye detection.

**Key points:**

*• LAMP assay enables large-scale screening of newborns for congenital CMV infection.*

*• LAMP allows colorimetric or quantitative detection of congenital CMV infection.*

*• LAMP assay can be used as a point-of-care testing tool in low-resource environments.*

**Supplementary Information:**

The online version contains supplementary material available at 10.1007/s00253-023-12771-2.

## Introduction

Cytomegalovirus (CMV) is one of the most common herpesvirus infections in humans (Moss 2020; Taylor 2003). Congenital CMV infection is transmitted through the placenta in a developing fetus (Pesch et al. 2021). This contrast with postnatal CMV infection, which is transmitted after birth through breast milk, saliva, or blood transfusion (Patel et al. 2019; Waters et al. 2019). The infection occurs in 0.5–1% of live births in developed countries and up to 5% of live births in developing countries (Akpan and Pillarisetty 2022; Chung et al. 2022; Marin et al. 2016; Pass and Arav-Boger 2018; Ssentongo et al. 2021; Zenebe et al. 2021). Congenital CMV infection is asymptomatic in 90% of newborns (Ronchi et al. 2020), with the remaining 10% of newborns exhibiting symptoms such as sensorineural hearing loss, and neurological impairment, which can lead to death (Bristow et al. 2011; Rahav et al. 2007). However, approximately 10% of asymptomatic newborns will also develop sensorineural hearing loss (Boppana et al. 2013).

A standard universal neonatal screening procedure has not yet been developed for CMV. Currently, congenital CMV diagnosis testing is performed via polymerase chain reaction (PCR) only when newborns exhibit symptoms, such as a hearing problem or rash (Lazzarotto et al. 2020). As a result, most asymptomatic newborns are not diagnosed with congenital CMV infection. Therefore, researchers are increasingly calling for a screening procedure to enable the timely diagnosis of congenital CMV infection in newborns (Cannon et al. 2014; Chiopris et al. 2020; Marsico and Kimberlin 2017; Ronchi et al. 2017; Tastad et al. 2019). Congenital CMV infection is typically confirmed by detecting virus DNA in blood, urine, or saliva by PCR within 3 weeks of birth (Plosa et al. 2012) since it is impossible to distinguish between congenital and acquired CMV infection thereafter. Unfortunately, serological CMV tests capturing the IgM and IgG antibodies of CMV cannot determine congenital CMV infection as both antibodies are produced at least 1 or 2 weeks after CMV infection (Fan et al. 2017; Iijima 2022). Although several types of PCR have been employed to diagnose congenital CMV (Leruez-Ville et al. 2011; Paixao et al. 2012; Pellegrinelli et al. 2019), a gold standard for screening congenital CMV does not yet exist (Pellegrinelli et al. 2020).

Loop-mediated isothermal amplification (LAMP) is a simple, rapid, and cost-effective nucleic acid amplification method (Tomita et al. 2008), in which six primers are employed to efficiently amplify a target DNA sequence under isothermal conditions. As the DNA is amplified, loop structures of amplicons are generated, leading to further amplification (Notomi et al. 2000). As LAMP results can be observed colorimetrically with the naked eye, costly instruments such as a thermocycler are not required; thus, the method can be used for point-of-care testing in low-resource environments. In this study, we used the LAMP assay to quantify CMV viral load in a sample, then compared the results with those of conventional quantitative PCR (qPCR), and evaluated the feasibility of LAMP assay for detecting congenital CMV infection.

## Materials and methods

### Conventional qPCR

A Real-Q CMV Quantification Kit (BioSewoom Inc., Seoul, Republic of Korea) was used to perform conventional qPCR for detecting and quantifying CMV. The kit, which has been approved by the Korean Ministry of Food and Drug Safety, consists of Taqman reagents and a probe that targets the DNA sequence corresponding to CMV envelope glycoprotein B (UL55) (Bae et al. 2013; Park et al. 2016). Conventional qPCR was performed using the QuantStudio™ 6 Flex Real-Time PCR System (Thermo Fisher Scientific Inc., MA, USA) according to the manufacturer’s instructions.

### Positive control

For the positive control, the *UL75* (glycoprotein H) sequence of the CMV Merlin strain (GenBank accession number AY446894) was used. The *UL75* nucleotide sequence was obtained from GenBank (http://www.ncbi.nlm.nih.gov/genbank/) and inserted into a pBHA vector (Bioneer Inc., Daejeon, Republic of Korea). The concentration was measured using NanoDrop 2000 (Thermo Fisher Scientific Inc., MA, USA), and the sequence was diluted for use as a positive control.

### Primer design

We used PrimerExplorer V5 software (http://primerexplorer.jp/lampv5e/index.html) to design three sets of LAMP primers (Primer ID: UL75, UL75-2, and UL75-3), which are complementary to the *UL75* sequence of CMV. These LAMP primers consisted of six primers including loop primers. A LAMP primer mix (2 μM F3, 2 μM B3, 16 μM forward inner primers, 16 μM backward inner primers, 4 μM LF, and 4 μM LB) was prepared prior to experimentation. LAMP primers are shown in Fig. S1.

### LAMP assay validation

#### Quantitative LAMP (qLAMP) assay

qLAMP reactions were conducted using WarmStart® LAMP 2 × Master Mix (DNA and RNA) (New England Biolabs® Inc., MA, USA). A reaction mixture (25 μL) was prepared by adding 12.5 μL of LAMP Master Mix (2 ×), 0.5 μL of LAMP Fluorescent Dye (50 ×), 0.5 μL of ROX Reference Dye (50 ×), 2 μL of LAMP primer mix, and 1 μL of template DNA, with DNase-free water used to make up the final volume of 25 μL. Template DNA was diluted tenfold from 1.1 × 10^8^ to 1.1 × 10^2^ copies/μL. Isothermal amplification was performed for 55 s at 66 ℃ for 40 cycles, followed by melting curve analysis (60 ℃ to 95 ℃ with 0.2 ℃/s increments).

#### Colorimetric LAMP assay

Colorimetric LAMP reactions were performed using the WarmStart® Colorimetric LAMP 2X Master Mix (DNA and RNA). A reaction mixture (25 μL) was prepared by adding 12.5 μL of LAMP Master Mix (2 ×), 2 μL of LAMP primer mix, 1 μL of template DNA, and DNase-free water to make up the final volume of 25 μL. The mixture was then incubated using a PCR thermal cycler (Takara Bio Inc., Shiga, Japan).

### Robustness of the LAMP assay

#### Applicability

To estimate the applicability of the colorimetric LAMP assay, we conducted LAMP assay experiments using four different heat sources: PCR machine (QuantStudio™ 6 Flex; Thermo Fisher Scientific Inc., MA, USA), thermocycler (Thermal Cycler Dice™ Touch; Takara Bio Inc., Shiga, Japan), thermomixer (ThermoMixer® C; Eppendorf Inc., Hamburg, Germany), and water bath (VS1205-W; Vision Scientific Co., Ltd., Daejeon, Republic of Korea).

#### Reproducibility and repeatability

The inter-rater correlation between operator 1 and operator 2 was analyzed using Pearson’s correlation coefficient (*r*) by comparing the Ct values of 12 different concentrations of positive controls. Similarly, the intra-rater reproducibility was evaluated between day 1 and day 2 with operator 1.

#### Analytical specificity

To estimate the analytical specificity of the LAMP assay, we performed cross-reactivity tests with six different herpes virus DNA controls (herpes simplex virus-1 (HSV-1), herpes simplex virus-2 (HSV-2), Epstein–Barr virus (EBV), varicella-zoster virus (VZV), human herpesvirus-6 (HHV-6), and human herpesvirus-8 (HHV-8)) by analyzing the amplification and melting curves.

### CMV viral load quantification in clinical samples

Clinical samples (*n* = 10) of urine were collected from newborns after obtaining consent from the parents. Subsequently, DNA was isolated from the urine samples, of which four were CMV-positive (CMV1, CMV2, CMV5, and CMV6) and the rest were CMV-negative (CMV3, CMV4, CMV7, CMV8, CMV9, and CMV10).

### Conversion between copies and international units

The 1st WHO International Standard for Human Cytomegalovirus for Nucleic Acid Amplification Techniques (NIBSC code 09/162) was used to convert viral load quantification units between copies and international units (IU). According to the manufacturer’s instructions, lyophilized CMV international standard was reconstituted to 5 IU$\times {10}^{6}$/mL with nuclease-free water. Then, 2 µL of CMV international standard was diluted 1:100 in a CMV-negative urine sample. Extraction was performed using the MagMAX™ Viral RNA Isolation Kit (Thermo Fisher Scientific Inc., MA, USA).

## Results

### Workflow of the LAMP assay

**Fig. 1:**
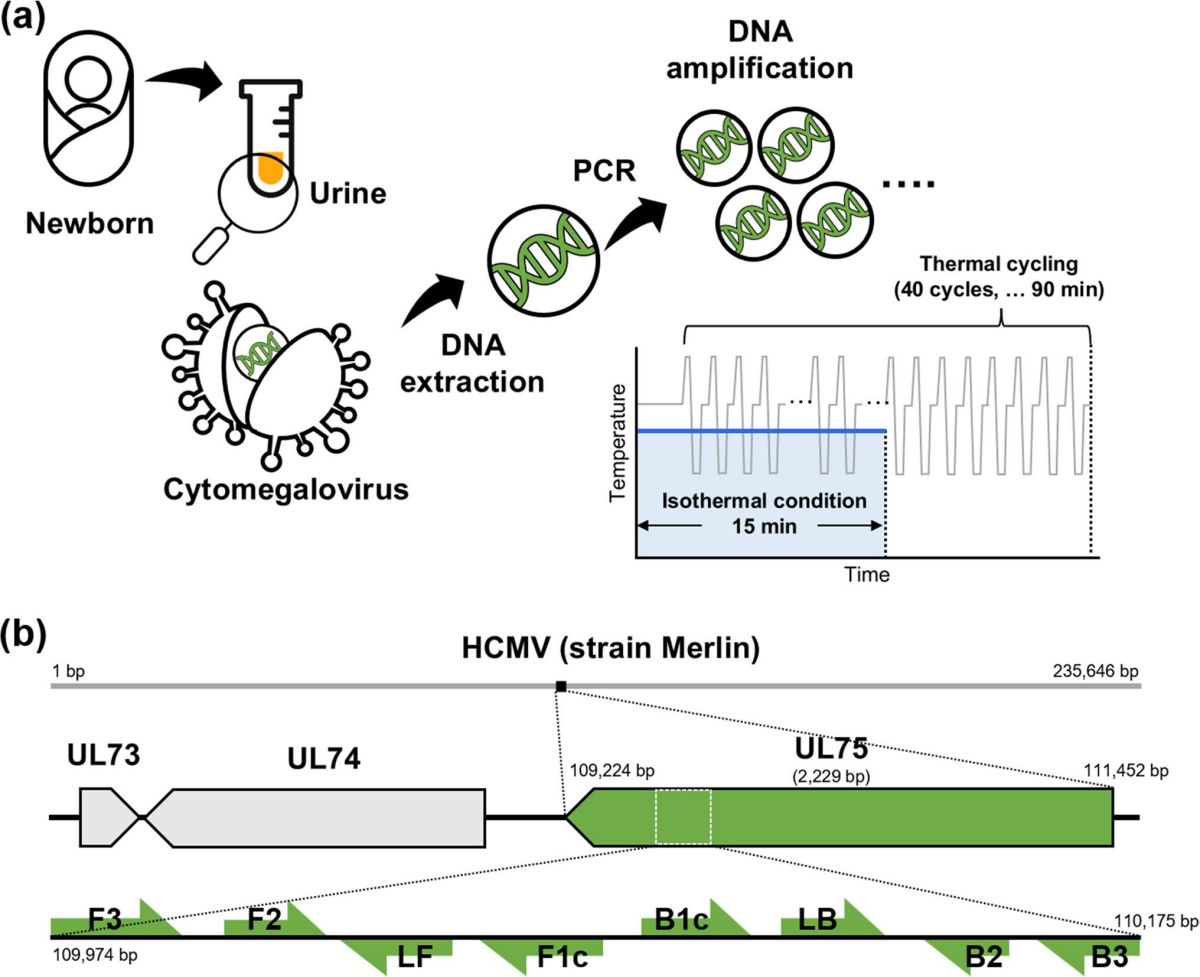
Loop-mediated isothermal amplification (LAMP) assay developed for cytomegalovirus (CMV) detection. **a** Schematic illustration of the workflow of congenital CMV screening in a newborn. CMV DNA is extracted from a newborn’s urine and then explosively amplified using six primers under isothermal condition. Forward inner primer consists of F2 and F1c primers, and backward inner primer consists of B2 and B1c primers. LAMP assay detected CMV within 15 min after extracting DNA from the urine sample, whereas the conventional qPCR method took more than 90 min after DNA extraction. **b** Location of *UL75* sequence and complementary LAMP primers targeting the *UL75* sequence

A workflow diagram of the LAMP assay is shown in Fig. 1a, and the location of the *UL75* sequence and its complementary LAMP primers is shown in Fig. 1b. Our study deals with the post-DNA extraction step. In conventional PCR, a temperature change is required in each cycle, which is a time-consuming process that leads to a prolonged turnaround time (TAT) of up to 90 min. In the LAMP assay, DNA is efficiently amplified under isothermal conditions; thus, the TAT was reduced to 15 min.

### Optimization of the LAMP assay

**Fig. 2:**
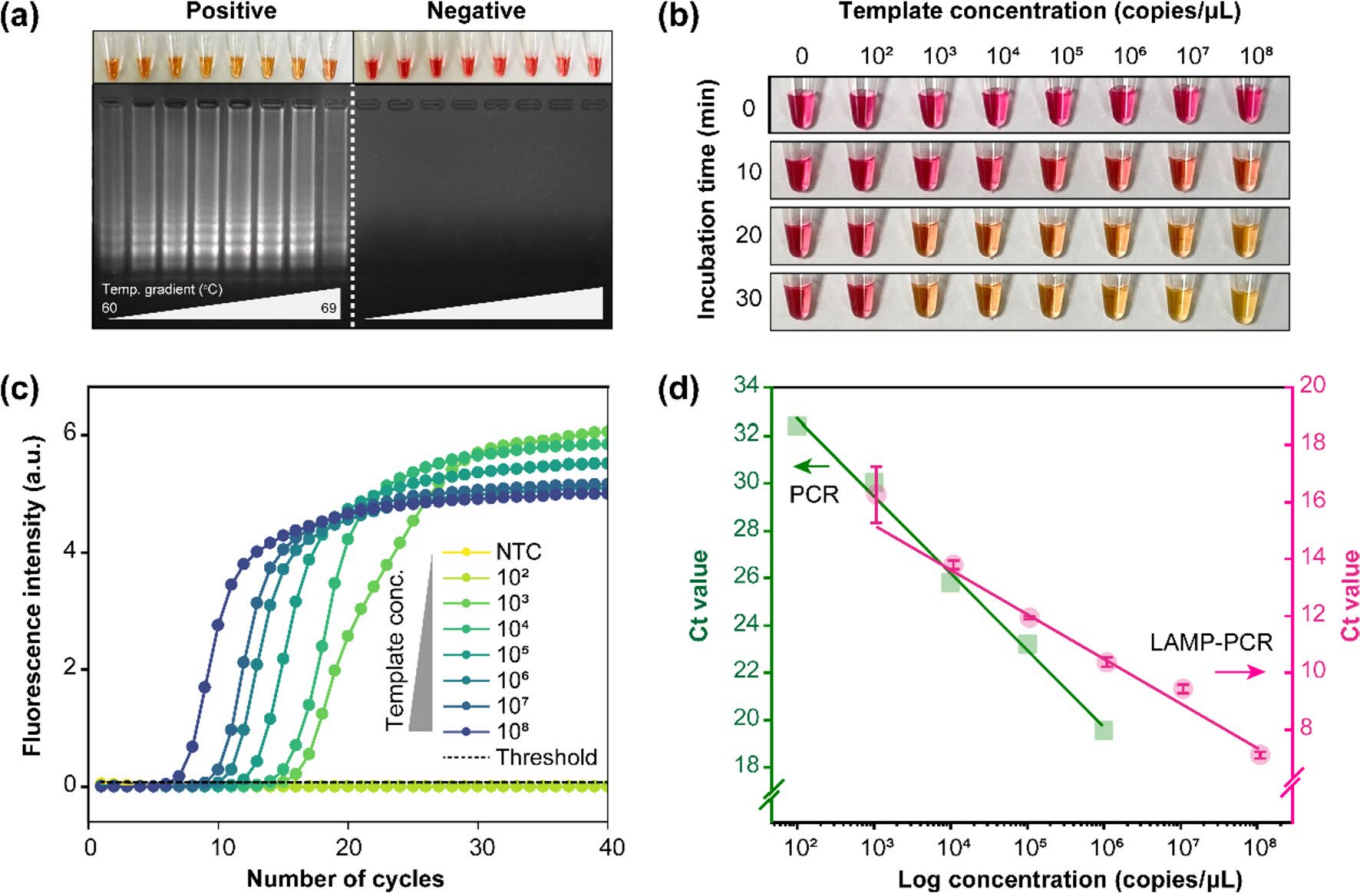
Validation of the loop-mediated isothermal amplification (LAMP) assay. **a** Optimization of the LAMP assay annealing temperature using eight different temperatures (60 ℃, 61 ℃, 62 ℃, 63.6 ℃, 64.8 ℃, 66.3 ℃, 67.5 ℃, and 69.1 ℃). Naked-eye detection of cytomegalovirus (CMV) by 2% agarose gel electrophoresis of LAMP reaction products (10 µL) following incubation at temperatures ranging from 60.3 to 69.1 ℃ for 30 min. A neutral pink color indicates a negative reaction, and a color change to yellow indicates a positive reaction. In positive reactions, 1.1 × 10^7^ copies/µL targets were present (lanes 1–8: positive reactions; lanes 9–16: negative reactions). **b** Naked-eye detection of tenfold diluted CMV DNA template (from 1.1 × 10^8^ to 1.1 × 10^2^ copies/µL) for different incubation durations (0, 10, 20, 30 min) at 66 ℃. **c** SYBR Green fluorescent read-out results of real-time LAMP amplification curve under tenfold diluted template from 1.1 × 10^8^ to 1.1 × 10^2^ copies/µL. The reactions were performed in quintuplicates, and the representative experiment out of five experiments was presented. **d** Standard curves from the amplification curve results. The pink straight line denotes standard curve of real-time LAMP assay. The reactions were performed in quintuplicates, and three out of five are expressed, excluding the minimum and maximum values. Standard deviation (SD) is represented with an error bar. The green straight line denotes standard curve of conventional qPCR

Colorimetric LAMP reactions were performed at eight different temperatures (60 ℃, 61 ℃, 62 ℃, 63.6 ℃, 64.8 ℃, 66.3 ℃, 67.5 ℃, and 69.1 ℃) for the naked-eye detection of CMV. Subsequently, to determine the optimal annealing temperature for the LAMP assay, we analyzed 10 µL of the LAMP reaction products via 2% agarose gel electrophoresis. The optimal annealing temperature results are shown in Fig. 2a, which indicates strong bands for temperatures from 62 to 67.5 ℃ and a lack of non-specific reactions. The results obtained with different sets of LAMP primers are also shown (Fig. S2). To confirm the optimal annealing temperature of the LAMP assay, we performed additional LAMP reactions at 62 ℃, 64 ℃, and 66 ℃. Subsequently, 66 ℃ was confirmed as the optimal annealing temperature for rapid CMV detection with the highest sensitivity (Fig. S3). Figure 2b shows the limit of detection of the LAMP assay at 66 ℃ for colorimetric naked-eye detection of CMV down to 1.1 × 10^3^ copies/µL within 20 min.

Figure 2c shows the LAMP amplification curves for the positive control. Amplification curves for the conventional qPCR method are also shown (Fig. S4). Figure 2d shows the standard curves of the amplification curves of the conventional qPCR method and the LAMP assay. The linear regression equation for the LAMP assay was derived from its standard curve, as follows:

$$y= -1.57622\mathrm{x }+ 19.93596$$

where *x* indicates the log concentration and *y* indicates the threshold cycle (Ct). The *y*-intercept denotes the cut-off value of the LAMP assay. Similarly, the linear regression equation for conventional qPCR method was derived from its standard curve as follows:

$$y= -3.24638\mathrm{x }+ 39.19014$$

Both linear regression equations were used to quantify the viral load in the samples.

### Robustness of the LAMP assay

**Fig. 3:**
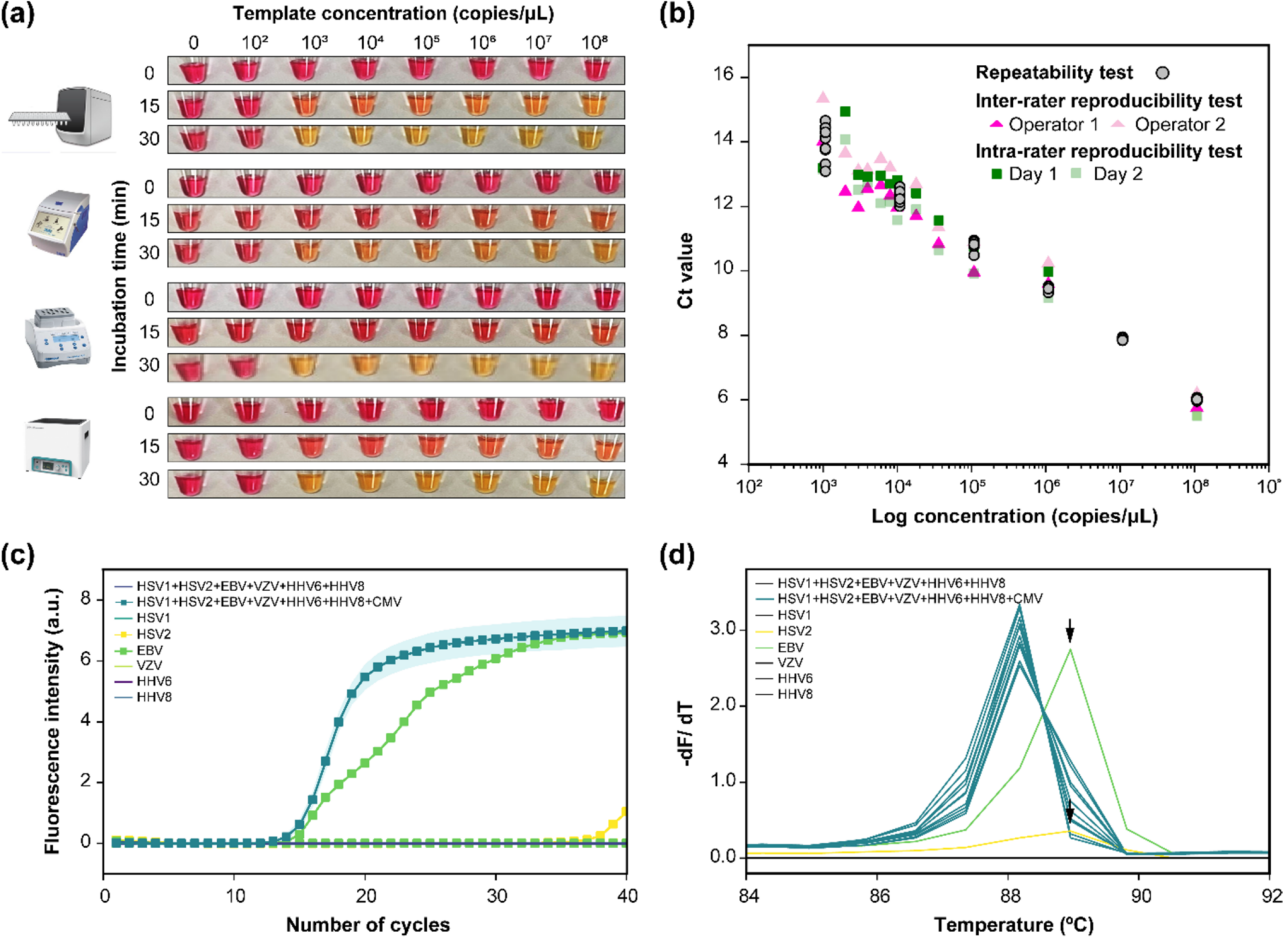
Robustness of the loop-mediated isothermal amplification (LAMP) assay. **a** Colorimetric naked-eye detection of CMV after incubation for 30 min with different heating instruments: PCR machine, thermocycler, thermomixer, and water bath. **b** Reproducibility and repeatability of the LAMP assay. Inter-rater reproducibility test was performed between operator 1 and operator 2 by comparing Ct values of 12 different concentrations of positive controls (1.0 × 10^3^, 2.0 × 10^3^, 3.0 × 10^3^, 4.0 × 10^3^, 6.0 × 10^3^, 8.0 × 10^3^, 1.0 × 10^4^, 1.8 × 10^4^, 3.6 × 10^4^, 1.1 × 10^5^, 1.1 × 10^6^, 1.1 × 10^8^ copies/µL). Similarly, intra-rater reproducibility test was performed between day 1 and day 2 with operator 1. Repeatability test was performed using tenfold serially diluted positive controls from 1.1 × 10^8^ copies/µL to 1.1 × 10^3^ copies/µL. The reactions were conducted 12 times at each concentration. Overall 132 Ct values from the reproducibility and repeatability tests were presented. Cross-reactivity analysis using six different herpes virus DNA controls (HSV-1, HSV-2, EBV, VZV, HHV-6, and HHV-8) with **c** amplification curve and the following **d** melting curve analysis. Amplification curve shows average of the 10 replicates of 1.0 × 10.^4^ copies/µL for all six different herpes virus ad CMV. Peaks indicated by arrows in the melting curve correspond to the dissociation curve of primer dimers that may form during the reaction. For the other samples that did not contain CMV, amplification curve of each sample is expressed as solid line. CMV, cytomegalovirus; HSV1, herpes simplex virus-1; HSV2, herpes simplex virus-2; EBV, Epstein–Barr virus; VZV, varicella-zoster virus; HHV6, human herpesvirus-6; HHV8, human herpesvirus-8

To evaluate the applicability of the LAMP assay, we performed a colorimetric LAMP assay with different heat sources. As shown in Fig. 3a, the LAMP assay gave consistent results, detecting CMV down to 1.1 × 10^3^ copies/µL within 30 min irrespective of the source of heat, indicating its potential for use as a point-of-care testing tool. Figure 3b shows the reliability of the LAMP assay. The inter-rater reproducibility test showed a strong positive correlation between operator 1 and operator 2, with *r* = 0.994. Moreover, the intra-rater reproducibility showed a strong positive correlation between day 1 and day 2 in operator 1, with *r* = 0.994. All 132 Ct values of the reliability tests (24 inter-rater reproducibility tests, 24 intra-rater reproducibility tests, and 84 repeatability tests) showed a positive linearity with *r* = 0.973. This suggests that the LAMP assay achieved consistent results and was not influenced by the operator, date of experiment, or number of replicates. According to Fig. 3c and d, the CMV target sequence in all 10 clinical samples was amplified, and these amplicons exhibited a melting temperature (*T*_m_) of 88.2 ℃. Two of the 70 samples (indicated by arrows) that tested negative for CMV exhibited non-specific amplification. One of these occurred in an EBV virus sample, while the other occurred in an HSV-2 virus sample. These samples were distinguished from those with specific amplification because they exhibited a higher *T*_m_ of 89.0 ℃. Furthermore, the LAMP assay in this study gave consistent results irrespective of the heat source.

### CMV viral load quantification in clinical samples

The PCR efficiency was calculated as follows:

$$E={10}^{-\left(\frac{1}{\mathrm{slope}}\right)}-1$$

Similarly, the number of replications per cycle was calculated as follows:

$$Number\;of\;replications\;per\;cycle={10}^{-\left(\frac{1}{\mathrm{slope}}\right)}$$

For example, the ideal slope of conventional PCR is − 3.32, from which the calculated PCR efficiency is 100%, and the number of replications per cycle is two. Then, the viral load is calculated as follows:

$$Viral\;load\;\left(copies/\mathrm{\mu L}\right) = {10}^{\frac{\left(\mathrm{Ct}-\mathrm{y\;intercept}\right)}{\mathrm{slope}}}$$

For viral load quantification, we performed DNA amplifications using both the conventional qPCR method and LAMP assay for 10 urine samples. The results are shown in Table 1. According to both qPCR and the LAMP assay, CMV1, CMV2, CMV5, and CMV6 were CMV-positive. Although CMV9 showed a Ct value of 26.957, it was denoted CMV-negative because its Ct value was higher than the predefined theoretical cut-off value of 19.94. Next, the CMV viral load was calculated using both qPCR and the LAMP assay. The quantification results differed by 1.59 times (CMV2) to 3.19 times (CMV 5) between the two methods. For CMV-positive samples, the average TAT for obtaining results using the LAMP assay was 13 min, whereas that using the conventional qPCR method was 59.5 min.

**Table 1:** CMV viral load quantification and turnaround time for obtaining amplification results after DNA extraction

	Conventional qPCR			LAMP		
Clinical samples	Ct	Viral load (copies/µL) (IU/mL)	Turnaround time (min)	Ct	Viral load (copies/µL) (IU/mL)	Turnaround time (min)
CMV1	26.107	10,716 (4.5 $\times$ ${10}^{6}$)	57.5	12.814	32,989 (2.75 $\times$ ${10}^{7}$)	12.2
CMV2	28.181	2,461 (1.0 $\times$ ${10}^{6}$)	61.1	14.908	1,548 (1.29 $\times$ ${10}^{6}$)	14.1
CMV3	-	-	-	-	-	-
CMV4	-	-	-	-	-	-
CMV5	25.848	12,878 (5.41 $\times$ ${10}^{6}$)	57.1	12.664	41,071 (3.42 $\times$ ${10}^{7}$)	12.0
CMV6	28.793	1,594 (6.70 $\times$ ${10}^{5}$)	62.2	14.219	4,243 (3.54 $\times$ ${10}^{6}$)	13.5
CMV7	-	-	-	-	-	-
CMV8	-	-	-	-	-	-
CMV9	-	-	-	26.957	N/A	N/A
CMV10	-	-	-	-	-	-
Average	27.232		59.5	13.651		13.0

- denotes too low to detect

## Discussion

Our study demonstrated that quantitative LAMP assay is rapid and cost-effective and can be used as a tool for newborn screening for congenital CMV infection.

Congenital CMV infection is a significant burden on both the health and economic systems. In the USA, the average annual cost of caring for children with disabilities resulting from congenital CMV infection, such as hearing loss and cognitive disabilities, was $30,000 USD per family between 2011 and 2016 (Meyers et al. 2019). In Germany, the estimated total lifetime cost for severe or profound hearing loss in a child is $0.28 million USD (Walter et al. 2018). In South Korea, the total healthcare costs, including reimbursement and patient co-payment, exceeded $80,000 USD per year according to data for 2010–2015 from the Health Insurance Review and Assessment Service (Choi et al. 2022). As a result, concerns about the need for effective screening for neonatal congenital CMV have been raised. In Italy, congenital CMV screening using the qPCR assay CMV ELITe MGB kit (ELITechGroup Molecular Diagnostics, Turin, Italy) was performed on the saliva samples of 3151 newborns born between February 2019 and July 2020. Only a 1.7% false-positive rate was observed (Chiereghin et al. 2022). In China, congenital CMV screening using qPCR was performed on the saliva or urine samples of 6350 newborns born between June 2015 and September 2017. The findings revealed that the combined testing of saliva and urine samples substantially improved the sensitivity of the results (Huang et al. 2021). However, it should be noted that such methods using conventional qPCR are expensive, time-consuming, and require expert know-how. In contrast, the qLAMP assay offers a more cost-effective alternative. A recent study provides evidence for the cost-effectiveness of qLAMP as a replacement for qPCR, with an average per-test cost of $8.45 for qLAMP and $14.75 for qPCR when considering all related costs (Iqbal et al. 2022). Similarly, our study revealed a cost per test of approximately $8.8 for the qPCR method, while the qLAMP assay demonstrated a significantly reduced cost of $2.4 per test, representing an impressive 73% reduction. Furthermore, the LAMP assay exhibits a significant advantage, as it can be performed colorimetrically without the need for costly instruments or quantitatively by combining it with a cost-effective fluorescence reader. This versatility allows for greater accessibility and applicability of the qLAMP assay in various laboratory settings. In the context of newborn screening, where timely testing is crucial before the baby reaches 3 weeks of age, the qLAMP assay excels in temporal efficiency, offering a significantly reduced testing time that is at least twice as fast as conventional qPCR methods. This rapid turnaround time enables healthcare providers to obtain the necessary results promptly, allowing for timely decision-making. Additionally, it is worth considering that in most countries, qPCR testing for congenital CMV infection is primarily focused on symptomatic newborns, which may limit its utility for early diagnosis of asymptomatic cases who may progress later. The qLAMP assay has the potential to broaden screening efforts, facilitating the detection of asymptomatic cases and enabling early diagnosis.

LAMP has shown significant promise as a screening method, especially in addressing pandemic situations and infectious diseases in resource-limited settings, such as SARS-CoV-2 (Huang et al. 2020; Kim et al. 2023, 2019), malaria (Ponce et al. 2017), human visceral leishmaniasis (de Avelar et al. 2019), and Zika virus (Silva et al. 2019). However, some challenges remain before it can replace conventional qPCR as a standard test. One of the limitations is the difficulty in achieving absolute quantification of the viral load in a sample. Some attempts have been made to quantify the viral load using LAMP, but these methods have relied on turbidity (Mori et al. 2004) or colorimetric naked-eye detection (Gonzalez-Gonzalez et al. 2021; Yu et al. 2021), which may not be sensitive enough for accurate quantification (Hardinge and Murray 2020; Nguyen et al. 2020). Additionally, other studies have shown that the amplification efficiency of LAMP may be inconsistent over time, which could also impact the accuracy of quantification (Lee et al. 2020; Thiessen et al. 2018). Our study provides preliminary evidence that LAMP may be capable of quantification, which has been validated using international standard. We observed a robust linear correlation between the Ct value and concentration, with a coefficient of determination (*R*^2^) of 0.98872. Additionally, our results demonstrated a larger dynamic range of detection compared to conventional qPCR, ranging from 1.1 × 10^3^ to 1.1 × 10^8^ copies/µL (9.2 × 10^5^ to 9.2 × 10^10^ IU/mL). This suggests that the assay replicates DNA approximately 4.31 times per cycle or 4.92 times per minute. On the other hand, some previous studies have raised concerns about non-specific amplification in the LAMP reaction, which can affect its reliability as a diagnostic tool (Jang and Kim 2022; Park 2022). To address this issue, additional melt curve analysis can be used to distinguish between target-specific and non-specific amplification (Chander et al. 2014). In our study, two samples showed non-specific amplification, but one sample was negligible and the other was further analyzed through cross-reactivity tests and nested PCR (Fig. S5). It was found that the non-specific amplification resulted from dimer formation of the LAMP primers. This finding is consistent with a previous study in which non-specific products with high *T*_m_ were found in the LAMP reaction and identified as a mixture of full-length forward and backward inner primers (Rolando et al. 2020).

In conclusion, we developed a qLAMP assay that enables the detection of neonatal congenital CMV both quantitatively and colorimetrically. Notably, our assay demonstrated a significantly broader dynamic range compared to conventional qPCR methods. We have highlighted the key advantages of our LAMP assay, including its shortened turnaround time and cost-effectiveness when compared to qPCR techniques. These findings underscore the potential of qLAMP as a valuable alternative for CMV detection and quantification in clinical settings.

### Supplementary Information

Below is the link to the electronic supplementary material.

Supplementary file1 (PDF 555 KB)

## Data Availability

The original contributions presented in this study are included in the article/supplementary material; further inquiries can be directed to the corresponding author.
