# Supplementary material for: Loop-mediated isothermal amplification assay for screening congenital cytomegalovirus infection in newborns
Source: Appl Microbiol Biotechnol. 2023 Sep 19;107(22):6789–98. doi: 10.1007/s00253-023-12771-2 (PMC10589182; doi:10.1007/s00253-023-12771-2)
Supplement: Supplementary file 1 — Supplementary file1 (PDF 555 KB) [file 253_2023_12771_MOESM1_ESM.pdf]

Loop-mediated isothermal amplification assay for screening congenital cytomegalovirus infection in newborns

Hyeonseek Park[1,2], Doo Ri Kim[3], Areum Shin[3], Eunjung Jeong[1,2], Sohee Son[3], Jin-Hyun Ahn[4], So Yoon Ahn[3], Suk-Joo Choi[5], Soo-young Oh[5], Yun Sil Chang[3], Yae-Jean Kim[3,6*], Minhee Kang[1,2*]

[1]Biomedical Engineering Research Center, Smart Healthcare Research Institute, Samsung Medical Center, Seoul, Republic of Korea

[2]Department of Medical Device Management and Research, Samsung Advanced Institute for Health Science & Technology, Sungkyunkwan University, Seoul, Republic of Korea

[3]Department of Pediatrics, Samsung Medical Center, Sungkyunkwan University School of Medicine, Seoul, Republic of Korea

[4]Department of Microbiology, Sungkyunkwan University School of Medicine, Suwon, Republic of Korea

[5]Department of Gynecology and Obstetrics, Samsung Medical Center, Sungkyunkwan University School of Medicine, Seoul, Republic of Korea

[6]Samsung Advanced Institute for Health Science & Technology, Sungkyunkwan University, Seoul, Republic of Korea

Correspondence

Yae-Jean Kim, MD, PhD

E-mail: yaejeankim@skku.edu

Minhee kang, PhD

E-mail: minhee.kang@samsung.com

Target Region	ID	Primer	Sequence	Amplicon (bp)
CMV_Merlin Strain UL75_941~1758	UL75	F3	CTCTAAACAGAATCAGCAACATCTC	202
		B3	GCCCGTTTCTACGATGAAGA	
		FIP	GGCTGAAAGAAAAGAGGCCAGGTG-CAATGGGCACTACGACAGAT	
		BIP	TCGCACGCCAAGAACTCTACC-TTCGCGTCTCTCCGTCG	
		LF	TAGTTTTAGGGCAAAGTCGGCG	
		LB	CAGCCTCGTCCACTCCATGC	
	UL75_2	F3	GCCGCACTCTTACAAATACAAGA	224
		B3	TCGGCGATCTGTCGTAGT	
		FIP	GCTCGTTTGGCCAGGTCCAC-TGATCACCTGCCTCTCACAA	
		BIP	CCGAATCAGATCACCGACATCACC-TTGGGGGATGAGATGTTGC	
		LF	AACGTGGTGCGTGGTGGTG	
		LB	TCGTACGCCTGGTCTACATACTCTC	
	UL75_3	F3	CAAGAATTTATGATCACCTGCCTC	189
		B3	TGCCCATTGGGGGATGA	
		FIP	TCGTTTGGCCAGGTCCACG-TCACAAACACCACCACGC	
		BIP	CCTTTGGACACCGAATCAGATCACC-GATGTTGCTGATTCTGTTTAGAGAG	
		LF	GGGATACAGCAGCAACGTG	
		LB	ATCACCAGCCTCGTACGCCT	

Fig. S1 Loop-mediated isothermal amplification (LAMP) primers sets information used in the LAMP assay. 3 candidate sets of LAMP primers were designed using PrimerExplorer V5 software (http://primerexplorer.jp/lampv5e/index.html).

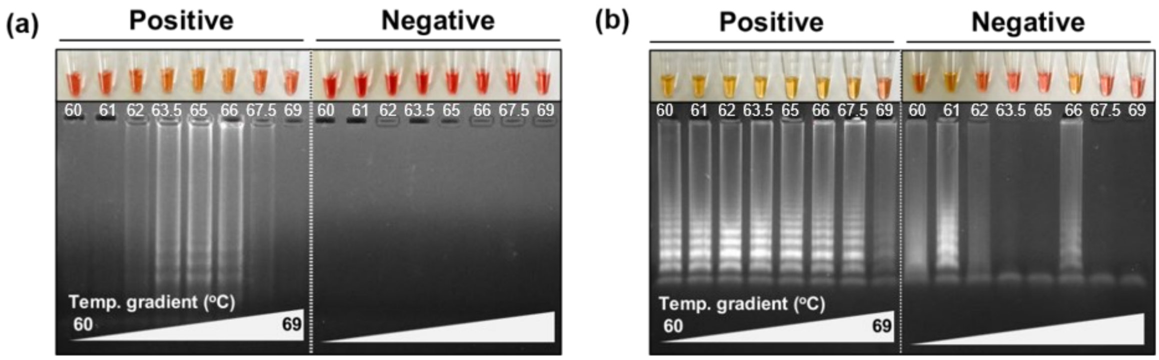

Fig. S2 Naked-eye detection of cytomegalovirus by 2% agarose gel electrophoresis of loop-mediated isothermal amplification (LAMP) reaction products (10 μL) with different sets of LAMP primers at temperatures ranging from 60.3 °C to 69.1 °C for 30 min. A neutral pink color indicates a negative reaction, and a color change to yellow indicates a positive reaction. In positive reactions, 1.1 × 10^7 copies/μL targets were present. (lanes 1–8: positive reactions; lanes 9–16: negative reactions). (a) UL75-2 LAMP primers set result. Relatively weak positive reactions were observed for temperatures between 62 °C and 67.5 °C, and non-specific reactions were not observed on negative reactions. (b) UL75-3 LAMP primers set result. Strong bands were observed over the entire annealing temperature, but non-specific reactions were also observed at the same time.

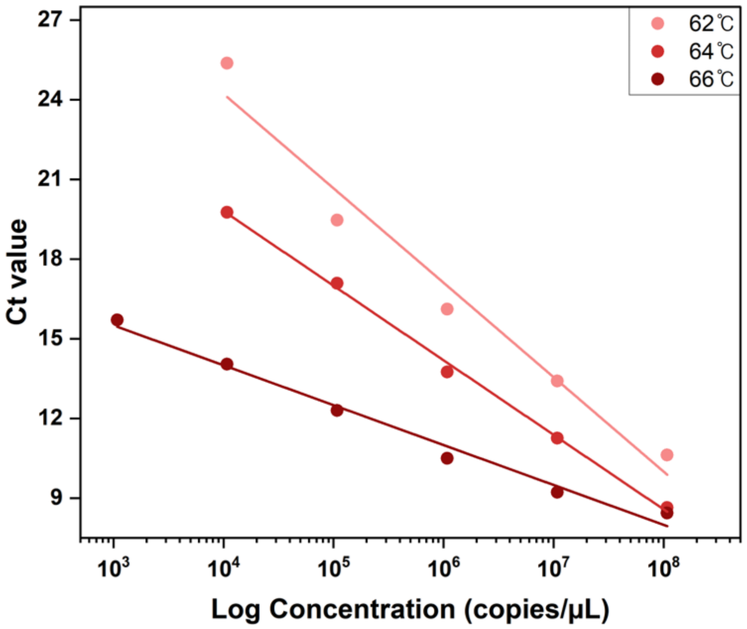

Fig. S3 Optimization of the loop-mediated isothermal amplification (LAMP) assay annealing temperature. LAMP reactions were performed at 62 °C, 64 °C, and 66 °C using 10-fold diluted DNA template ranging from 1.1 × 10^8 copies/μL to 1.1 × 10^2 copies/μL. LAMP assay at 62 °C and 64 °C detected CMV with dynamic range from 1.1 × 10^8 copies/μL to 1.1 × 10^4 copies/μL, and LAMP assay at 66 °C detected CMV with dynamic range from 1.1 × 10^8 copies/μL to 1.1 × 10^3 copies/μL. Linear regression equation from the standard curve is expressed as $y = -3.55x + 38.44$, $y = -2.81x + 31.03$, $y = -1.50x + 20.01$ at 62 °C, 64 °C, 66 °C, respectively.

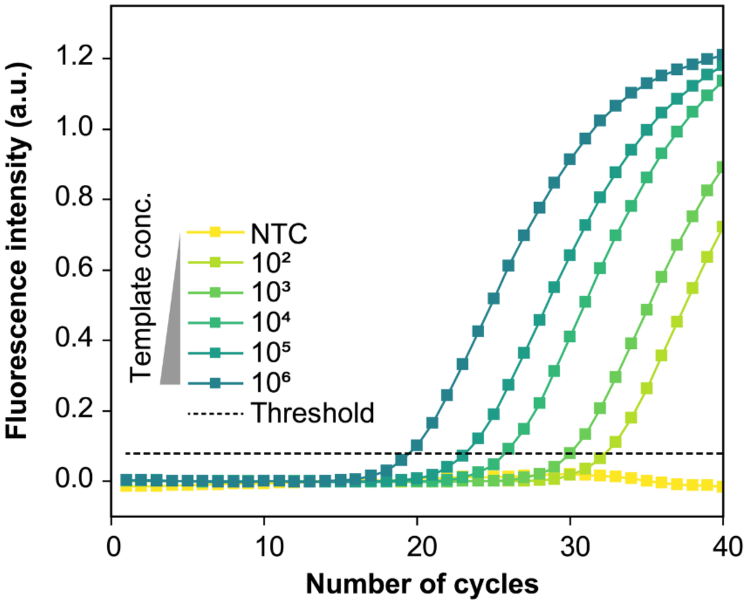

Fig. S4 Conventional quantitative PCR (Real-Q CMV Quantification kit; BioSewoom Inc, Seoul, Republic of Korea) amplification curves of 10-fold diluted DNA template (from 1.0×10^6 to 1.0×10^2 copies/µL)

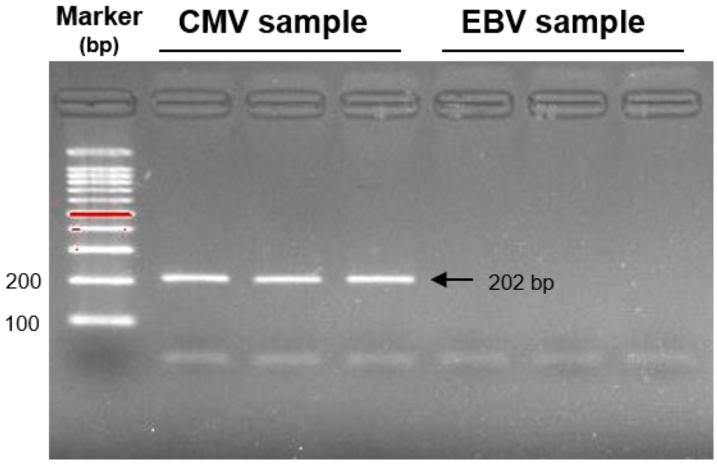

Fig. S5 Nested PCR of the non-specific amplification found in EBV sample results analyzed using 4% agarose gel electrophoresis. Nested PCR was performed in triplicate for both CMV-positive samples and EBV-non-specific amplification samples (lane 1: 100 bp DNA ladder; lanes 2–4: CMV positive reactions; lanes 5–7: non-specific amplification found in EBV samples). Nested PCR was conducted using Power SYBR® Green Master Mix (Thermo Fisher Scientific Inc., Massachusetts, United States). A reaction mixture (25 µL) was prepared by adding 12.5 µL of master mix, 2 µL of primer mix (consisting of 1 µL (10 µM) of F3 and 1 µL (10 µM) of B3 of the LAMP primer), 1:100 diluted products of the LAMP reaction as a target of the second PCR, and DNase-free water to make up the final volume of 25 µL. Amplification was performed for 10 min at 95 °C for first denaturation, followed by 40 cycles consisting of 15 s of denaturation at 95 °C, 20 s of annealing at 56 °C, and 20 s of extension at 72 °C. Reactions were conducted using the QuantStudio™ 6 Flex PCR machine.
